# Second-Hand Smoking among Intermediate and Secondary School Students in Madinah, Saudi Arabia

**DOI:** 10.1155/2015/672393

**Published:** 2015-07-26

**Authors:** Abdulmohsen H. Al-Zalabani, Soliman M. Amer, Khaled A. Kasim, Reem I. Alqabshawi, Ayat R. Abdallah

**Affiliations:** ^1^Family and Community Medicine Department, College of Medicine, Taibah University, Madinah 41541, Saudi Arabia; ^2^Department of Public Health and Community Medicine, Damietta Faculty of Medicine, Al-Azhar University, Damietta, Egypt; ^3^Public Health and Community Medicine Department, Faculty of Medicine, Al-Azhar University, Cairo, Egypt; ^4^Environmental Health of the Liver Department, National Liver Institute, Menoufia University, Shibin Al Kawm, Egypt

## Abstract

*Background and objectives*. Second-hand smoke (SHS) is an important public health problem worldwide. The study aimed to estimate the prevalence of SHS exposure and its associated risk factors among intermediate and secondary school students. *Methods*. A cross-sectional study was conducted in 2013 among 3400 students from 34 intermediate and secondary schools in Madinah City, Saudi Arabia. Data about sociodemographic and smoking-related factors and SHS exposure were collected using a self-administered questionnaire. *Results*. Of the 3210 students analyzed, the prevalence of SHS exposure was 32.7% 49.3%, and 25% inside, outside, and both inside and outside the home, respectively. The highest risk of SHS exposure was associated with the adolescent's smoking status, parental smoking, close friends smoking, and family structure. The risk was markedly increased in association with parental smoking for exposure inside the home (OR = 6.49; 95% CI = 5.44–7.73) and with close friends smoking for exposure outside the home (OR = 4.16; 95% CI = 3.54–4.77). The risk of SHS, however, was lower among adolescents having knowledge about smoking and highly educated parents. *Conclusion*. The study revealed a considerably high prevalence of SHS both inside and outside the home among adolescents. Knowledge and beliefs about SHS exposure are the main preventable approach.

## 1. Introduction

Second-hand smoke (SHS), which is also called environmental tobacco smoke (ETS), is the combination of side stream smoke given off by a burning tobacco product and mainstream smoke exhaled by a smoker [1, 2]. Exposure to SHS can cause heart disease and lung cancer in adults. In children, it is associated with increased risks for acute respiratory infections, middle ear disease, worsened asthma, respiratory symptoms, and slowed lung growth [2–4]. Research indicates there is no safe level of exposure to SHS [2]. Worldwide, 40% of children, 33% of male nonsmokers, and 35% of female nonsmokers were exposed to SHS, according to a 2004 WHO report [5]. In the Kingdom of Saudi Arabia (KSA), a national study conducted in 2008 reported a smoking prevalence of 36% and 3% among male and female adults, respectively [6].

Adolescence is a critical period characterized by psychological and behavioral changes that may affect adolescents' smoking behavior [7]. This makes intermediate and secondary school years a crucial period to study not only smoking prevalence and predictors but also the prevalence and risk factors of SHS during this period.

In developed countries, educating people about hazards of SHS was a focus area for health education efforts [8]. Article 8 of the WHO Framework Convention on Tobacco Control (FCTC) addresses the issue of protecting individuals from the dangers of SHS [9]. KSA is one of the countries that agreed with and became a part of the FCTC. It has implemented restrictions on tobacco use in enclosed places through ministerial decrees. The restrictions include 100% smoke-free hospitals and health care facilities, educational facilities, and governmental facilities [10]. Previous studies in the region showed low awareness about policies related to the framework convention [11], which highlight importance of raising awareness to enhance control efforts.

The Global Youth Tobacco Survey (GYTS) worldwide data indicated that approximately 47% of adolescents who never smoked were exposed to SHS at home, and approximately 48% were exposed to SHS outside households. Those who have been exposed to SHS at home were more likely to initiate smoking than those who were not exposed. Adolescents exposed to SHS in places other than home were more likely to start smoking than those not exposed [12]. In KSA, about 29.5% of adolescents were exposed to SHS at home, and approximately 37.5% were exposed to SHS outside households. Exposure to SHS significantly impacts the lives of people throughout the Eastern Mediterranean Region, as evidenced by a recent retrospective review of the burden of disease [5]. There is a scarcity of data about the prevalence and risk factors of SHS exposure among adolescents in Saudi Arabia. This study aimed to estimate the prevalence of SHS exposure at home and outside the households and to investigate the possible associated risk factors among intermediate and secondary school students in Almadinah Almunawwarah (Madinah) city, Saudi Arabia.

## 2. Methods

This school-based, cross-sectional study analyzed data collected from 3210 students from intermediate and secondary schools in Madinah City, Saudi Arabia, during the period from the first of January to the first of May of 2013. A multistage, stratified cluster sampling procedure was employed in which schools in Madinah city were divided into strata according to their levels (intermediate versus secondary) and students' sex (male versus female), with the final sample being proportional to the size of the stratum. Within each stratum, cluster sampling was implemented in which the primary sampling unit was the school. Finally, within each selected school, one class from each grade was randomly selected, where all students in the selected class were invited to participate.

The study data were collected through a structured, self-administered, anonymous questionnaire. The questionnaire was based on the Global Youth Tobacco Survey (GYTS) questionnaire [13]. The questionnaire was translated to Arabic and verified by back-translation performed by a different bilingual person. The face validity of the Arabic questionnaire was discussed with public health and tobacco control experts.

The primary calculated sample of this study was 780 students based on averages of the estimated smoking prevalence among school students in previous Saudi studies (20–30%), an assumed precision of 3% and a confidence interval of 95%. To obtain the same level of accuracy in both male and female students as well as in intermediate and secondary schools, the sample size was quadrupled to 3120 students. Accounting for nonresponse, the sample size increases to 3400 students.

Trained public health personnel briefed the students about the study and their participation and asked them to complete the study questionnaire anonymously after giving their consent. The school officials were clearly informed about the aim and scope of the study. Students were informed that participation was voluntary. The confidentiality and privacy of the collected data were ensured throughout the study. Finally, the study protocol was approved by the Ethics Committee at College of Medicine, Taibah University.

Second-hand smoke exposure (the dependent variable) was assessed in the study questionnaire by the following questions: “during the past 7 days, on how many days has anyone smoked inside your home, in your presence?” and “during the past 7 days, on how many days has anyone smoked in your presence, in a public place other than your home?” Exposure to SHS was defined as being exposed to SHS at least one day in the past 7 days, while nonexposure was having never been exposed to SHS in the past 7 days. Finally, the SHS exposure variable was categorized into three categories: at home exposure only, outside households exposure only, and overall exposure. Outside household exposure included SHS exposure in school, public places, social clubs, playgrounds, Internet cafes (Cybercafés), parks, and restaurants.

The independent variables included in this study were (i) sociodemographic characteristics, including age in years, sex (male versus female), school level (intermediate versus secondary), mother's and father's education (no formal education, less than university, university, and higher), and living status (lives with one or both parents versus lives with neither) and (ii) smoking-related factors, including smoking status (smoker versus nonsmoker), parental smoking (none versus one or both parents smoke), close friends smoking (none versus some or all friends smoke), knowledge about smoking from family or school (yes versus no), and belief in the negative effects of smoking (yes versus no).

The data were analyzed using the Statistical Package for the Social Sciences (SPSS) version 16 [14]. Descriptive statistics were used to estimate the prevalence of SHS, and the chi-square test was used to compare exposed and nonexposed groups by socioeconomic variables. *P* values ≤ 0.05 were considered as evidence of statistical significance. Multivariate logistic regression analyses were used to investigate the association between SHS exposure and the possible risk factors.

## 3. Results

The overall response rate in this study was 97.7% (3322/3400), with no significant school-level difference. The response rate was 97.5% (1561/1600) in intermediate schools and 97.8% (1761/1800) in secondary schools. Because of missing data related to the studied variables, a total of 3210 students were included in the study analyses. The prevalence of SHS exposure among the studied adolescents was 32.7%, 49.3%, and 25% at home, outside households, and overall exposure, respectively.

Table 1 presented the characteristics of the studied adolescents by sociodemographic variables and SHS exposure. There were highly significant differences between exposed and nonexposed adolescents regarding nearly all sociodemographic variables. The highest percentage of exposure was among adolescents living with neither parent (71.4%), age >16 years (64.6%), male (62.1%), and secondary school students (62.7%). Also, the highest frequency of SHS exposure was among adolescents whose fathers have no formal education (61.1%).

Table 2 presents adjusted odds ratios and their 95% confidence intervals for the association between sociodemographic and smoking-related variables and overall SHS exposure (at home and outside household exposures). The highest odds of exposure were associated with parental smoking (OR = 4.95; 95% CI = 4.15–5.90), close friends smoking (OR = 3.71; 95% CI = 3.09–4.33), smoking status of the adolescent (OR = 3.66; 95% CI = 3.06–4.27), and living with neither parent (OR = 2.66; 95% CI = 1.75–4.05). Adolescents' age, sex, and school level were also associated with increased odds of exposure. On the other hand, however, the risk was reduced among adolescents with highly educated parents and those having knowledge about smoking and belief in the harmful effects of SHS.

Table 3 presents the risk of adolescents' exposure to SHS at home. Parental smoking was associated with the highest odds of SHS exposure, with an adjusted OR of 6.49 (95% CI = 5.44–7.73). The smoking status of the adolescent, close friends smoking, and living with neither parent were also associated with increased odds of exposure to SHS at home, with an adjusted OR of 3.11 (95% CI = 2.66–3.63), 2.75 (95% CI = 2.36–3.20), and 2.26 (95% CI = 1.48–3.42), respectively. Parents' education, particularly education of the father, was associated with significantly decreased odds of SHS exposure at home. The risk was reduced, however, among adolescents with a highly educated father and among adolescents with beliefs about the harm of SHS.

Table 4 presents the risk of adolescents' exposure to SHS outside household. The main factors were associated with increased odds of SHS exposure outside household. The odds were increased 4-fold among adolescents who were smokers (OR = 4.12; 95% CI = 3.34–4.60) and those having close friends smoking (OR = 4.16; 95% CI = 3.54–4.77). Other factors found to increase the odds of exposure were parental smoking, age >16 years, living with neither parent, being secondary school students, and male sex. The risk of exposure, however, was significantly reduced among adolescents having knowledge about the harmful effects of smoking, with an adjusted risk of 0.68 (95% CI = 0.58–0.84).

## 4. Discussion

The prevalence of adolescents' exposure to SHS in Madinah, Saudi Arabia, was 32.7% at home and 49.3% outside households, with the overall SHS exposure being 25%. A similar high prevalence of adolescent SHS was also reported in a recent study conducted in Hong Kong [15], in which the prevalence of SHS exposure was 42% among the studied adolescents. The GYTS reported that more than half of the surveyed students worldwide have been exposed to SHS in public places. In the Eastern Mediterranean region, exposure to SHS was 38% inside homes and 46% in public places [16]. In a recent study conducted in Riyadh, Saudi Arabia, the prevalence recorded was 27.9% for exposure at home and 38.2% for exposure outside household [17]. The higher prevalence of exposure at home and outside household compared to that in the Riyadh study may be attributed to different locations or may be because the Riyadh study has included young adolescent intermediate school students.

In this study, the majority of those in the exposed group were males (55.8%), compared with 44.2% of females. The same result was found in the Riyadh study, where males were exposed to SHS (either inside or outside the home) more than females. Regarding education of the father, the highest frequency of exposure was among those adolescents whose fathers have no formal education (61.1%) and those with less than university educated mothers (58.4%). These findings coincided with the results of a survey study conducted on Malaysian children [18], which found that the highest concentration of salivary cotinine (resulting from exposure to SHS) was among children with a middle-school-educated father, and the lowest percentage was for those with a university educated father.

The present study revealed that the highest risk of exposure at home and outside household was associated with parental smoking, close friends smoking, the smoking status of the adolescent, and living with neither parent. A marked risk was observed with parental smoking for SHS exposure at home and with close friends smoking for SHS exposure outside household. Nearly the same results were found in the Raute et al. study [19]. They ranked the risk factors associated with SHS exposure at home as follows: parental smoking (OR = 9.40; 95% CI = 5.32–16.41), followed by close friends smoking (OR = 2.93; 95% CI = 2.20–3.89), and, finally, the smoking status of the studied adolescents (OR = 2.11; 95% CI = 1.33–3.34).

The odds of adolescent exposure to SHS and specifically outside household were significantly increased among late adolescents (>16 years). Nearly the same results were found by Raute et al. [19], who reported that the age range of 15 to 17 years poses a higher risk for SHS exposure both at home and outside household. Males were also found in our study to have a higher risk of exposure to SHS and specifically to SHS outside household.

Secondary school students were at a higher risk of exposure to SHS both at home and outside household. This finding may be attributed to the higher ages of the secondary school students and more freedom given to them compared with young intermediate school children. The adolescents living with neither parent were at increased odds for overall SHS exposure.

In the present study, the risk of SHS exposure outside household was decreased among adolescents having knowledge about the harmful effects of smoking compared with those having no knowledge. The association between knowledge about smoking health hazards and the risk of SHS exposure was not addressed properly in previous studies, but knowledge was measured among adolescents in some studies [13] and showed that the median percent of students who reported having been taught in school about the dangers of smoking was 50.8%, ranging from 83% in China to less than 30% in some states in India. In Saudi Arabia, a GYTS study conducted in 2007 [17] reported that 58.8% of surveyed students (66.1% among females and 52.9% among females) had been taught in their schools about the dangers of smoking.

The present study revealed a 25% reduction in the risk of exposure to SHS at home among adolescents believing that smoking is harmful. In a Mumbai study [19], there were increased odds of SHS exposure at home among adolescents who had no awareness about the harmful effects of SHS. The perception of harmfulness of exposure to SHS was addressed also in another study [20] as a protective factor for adolescents' initiation of smoking.

The present study has a number of strengths that include being school-based with a relatively large sample size and high response rate among the interviewed students, which supports the robustness of the study findings. Furthermore, the study addressed SHS exposure both at home and outside household. Finally, the results presented in this study were precisely estimated, as indicated by the observed narrow confidence intervals.

However, the limitations of this study should not be overlooked. The data collection in the study was based on self-completion of the GYTS questionnaires. The validation of self-report via biochemical tests was not feasible due to logistical and cultural constraints.

In summary, although Saudi Arabia is considered one of the pioneer tobacco control countries in the region, this study revealed a considerably high prevalence of adolescents being exposed to SHS in Madinah City. One of the main findings of this study was the decreasing risk effect of knowledge about the harmful effects of smoking and beliefs of adolescents that SHS is very harmful. This signifies the need for schools and families to increase awareness of their students towards the hazards of SHS exposure.

The risk of SHS exposure in this study was related to the adolescents' and parents' smoking status. This finding reflects the need to design an appropriate and effective antismoking education program addressing smoking predictors and targeting not only school students but also their friends, families, and school members. SHS exposure is a risk factor to start smoking especially in this critical period in adolescents' life.

Also, the study findings provide a significant alarm to the country authorities regarding the need to adopt more preventive strategies in addition to the present smoking legislation. Adolescents are exposed to SHS inside and outside home, so parental education is of paramount importance. Health advocacy toward adopting smoke-free policies for homes, restaurants, and coffee shops is highly needed. The existing activities conducted in schools in collaboration with tobacco control governmental agencies as well as nonprofit organizations need to be augmented by formal training programs for teachers with outcome monitoring. Finally, in such Moslem communities, mosques appeared to have a crucial role in raising the awareness of youth and their families not only on the harmful effect of smoking, but also on religion's stance on this habit.

## Figures and Tables

**Table 1:** Characteristics of the studied adolescents by sociodemographic variables and SHS exposure.

Variable	SHS exposure				*P* value
	Exposed		Not exposed		
	Number	%	Number	%	
Exposure					
Outside household	1049	32.7	2161	67.3	
At home	1584	49.3	1626	50.7	
Overall	801	25.0	2409	75.0	
Smoking status					
Nonsmoker	365	16.8	1811	83.2	0.00^∗^
Smoker or former smoker	436	42.2	598	57.8	
Age (in years)					
<13	213	45.4	256	54.6	0.00^∗^
13–16	919	55.5	738	44.5	
>16	700	64.6	384	35.4	
Sex					
Female	809	51.8	754	48.2	0.00^∗^
Male	1023	62.1	624	37.9	
School grade					
Intermediate	912	52.3	831	47.7	0.00^∗^
Secondary	920	62.7	547	37.3	
Father's education					
No formal education	96	61.1	61	38.9	0.01^∗^
Less than university	833	59.6	564	40.4	
University or higher	903	54.5	753	45.5	
Mother's education					
No formal education	127	55.5	102	44.5	0.33
Less than university	931	58.4	664	41.6	
University or higher	774	55.8	612	44.2	
Family structure					
One or both parents	1767	56.7	1352	43.3	0.00^∗^
Neither	65	71.4	26	28.6	

^∗^Significant difference.

**Table 2:** Adjusted odds ratios and their 95% confidence intervals (CIs) for the association between sociodemographic and smoking-related variables with overall SHS exposure.

Factor	Overall SHS exposure		OR^∗^	95% CI
	Exposed *N* = 801	Not exposed *N* = 2409		
Age (in years)				
<13	91	378	**1.00**	**Reference**
13–16	415	1242	1.43	1.24–1.61
>16	295	789	1.61	1.14–2.25
Sex				
Female	377	1186	**1.00**	**Reference**
Male	424	1223	1.14	0.92–1.28
School grade				
Intermediate	398	1345	**1.00**	**Reference**
Secondary	403	1064	1.33	1.09–1.50
Father's education				
No formal education	49	108	**1.00**	**Reference**
Less than university	377	1020	0.76	0.67–0.98
University or higher	375	1281	0.65	0.50–0.87
Mother's education				
No formal education	67	162	**1.00**	**Reference**
Less than university	389	1206	0.82	0.66–1.17
University or higher	345	1041	0.85	0.71–1.20
Family structure				
One or both parents	759	2360	**1.00**	**Reference**
Neither	42	49	2.66	1.75–4.05
Smoking status				
Nonsmoker	365	1811	**1.00**	**Reference**
Smoker or former smoker	436	598	3.66	3.06–4.27
Parents smoking				
Nonsmokers	408	2017	**1.00**	**Reference**
One or both are smokers	393	392	4.95	4.15–5.90
Close friends smoking				
No	265	1552	**1.00**	**Reference**
Some of them smoke	536	857	3.71	3.09–4.33
Knowledge about harmful effects of smoking				
No	593	1731	**1.00**	**Reference**
Yes	208	678	0.89	0.75–1.07
Belief in harmful effects of SHS				
No	311	791	**1.00**	**Reference**
Yes	490	1618	0.71	0.65–0.90

^∗^Each variable is adjusted by the other variables in the table.

**Table 3:** Adjusted odds ratios and their 95% confidence intervals (CIs) for the association between sociodemographic and smoking-related variables and SHS exposure at home.

Factor	SHS exposure at home		OR^∗^	95% CI
	Exposed *N* = 1049	Not exposed *N* = 2161		
Age (in years)				
<13	128	341	**1.00**	**Reference**
13–16	555	1102	1.33	0.96–1.49
>16	366	718	1.36	0.98–1.52
Sex				
Female	520	1043	**1.00**	**Reference**
Male	529	1118	0.95	0.82–1.10
School grade				
Intermediate	544	1199	**1.00**	**Reference**
Secondary	505	962	1.16	0.99–1.34
Father's education				
No formal education	64	93	**1.00**	**Reference**
Less than university	497	900	0.75	0.66–0.86
University or higher	488	1168	0.53	0.42–0.69
Mother's education				
No formal education	84	145	**1.00**	**Reference**
Less than university	527	1068	0.95	0.74–1.15
University or higher	438	948	0.84	0.62–1.11
Family structure				
One or both parents	1002	2117	**1.00**	**Reference**
Neither	47	44	2.26	1.48–3.42
Smoking status				
Nonsmoker	531	1645	**1.00**	**Reference**
Smoker or former smoker	518	516	3.11	2.66–3.63
Parents smoking				
Nonsmokers	539	1886	**1.00**	**Reference**
One or both are smokers	510	275	6.49	5.44– 7.73
Close friends smoking				
No	419	1398	**1.00**	**Reference**
Some of them smoke	630	763	2.75	2.36–3.20
Knowledge about harmful effects of smoking				
No	780	1544	**1.00**	**Reference**
Yes	269	617	0.87	0.73–1.02
Belief in harmful effects of SHS				
No	393	709	**1.00**	**Reference**
Yes	656	1452	0.75	0.69–0.95

^∗^Each variable is adjusted by the other variables in the table.

**Table 4:** Adjusted odds ratios and their 95% confidence intervals (CIs) for the association between sociodemographic and smoking-related variables and SHS exposure outside household.

Factor	SHS outside households		OR^∗^	95% CI
	Exposed *N* = 1584	Not exposed *N* = 1626		
Age (in years)				
<13	176	293	**1.00**	**Reference**
13–16	779	878	1.55	1.12–1.74
>16	629	455	2.42	1.74–2.85
Sex				
Female	666	897	**1.00**	**Reference**
Male	918	729	1.70	1.40–2.07
School grade				
Intermediate	766	977	**1.00**	**Reference**
Secondary	818	649	1.74	1.39–1.85
Father's education				
No formal education	81	76	**1.00**	**Reference**
Less than university	713	684	0.92	0.74–1.26
University or higher	790	866	0.88	0.72–1.07
Mother's education				
No formal education	110	119	**1.00**	**Reference**
Less than university	793	802	1.01	0.66–1.79
University or higher	681	705	0.99	0.50–1.24
Family structure				
One or both parents	1524	1595	**1.00**	**Reference**
Neither	60	31	2.12	1.30–3.14
Smoking status				
Nonsmoker	846	1330	**1.00**	**Reference**
Smoker or former smoker	738	296	4.12	3.34–4.60
Parents smoking				
Nonsmokers	1076	1349	**1.00**	**Reference**
One or both are smokers	508	277	2.29	1.94–2.71
Close friends smoking				
No	629	1188	**1.00**	**Reference**
Some of them smoke	955	438	4.16	3.54–4.77
Knowledge about harmful effect of smoking				
No	1207	1117	**1.00**	**Reference**
Yes	377	509	0.68	0.58–0.80
Belief in harmful effects of SHS				
No	530	572	**1.00**	**Reference**
Yes	1054	1054	1.08	0.93–1.24

^∗^Each variable is adjusted by the other variables in the table.
